# Genome-wide analysis of the TPX2 family proteins in *Eucalyptus grandis*

**DOI:** 10.1186/s12864-016-3303-0

**Published:** 2016-11-24

**Authors:** Pingzhou Du, Manoj Kumar, Yuan Yao, Qiaoli Xie, Jinyan Wang, Baolong Zhang, Siming Gan, Yuqi Wang, Ai-Min Wu

**Affiliations:** 1 State Key Laboratory for Conservation and Utilization of Subtropical Agro-Bioresources, South China Agricultural University, Guangzhou, 510642 China; 2 Guangdong Key Laboratory for Innovative Development and Utilization of Forest Plant Germplasm, College of Forestry and Landscape Architecture, South China Agricultural University, Guangzhou, 510642 China; 3 College of Life Sciences, South China Agricultural University, Guangzhou, 510642 China; 4 Provincial Key Laboratory of Agrobiology, Jiangsu Academy of Agricultural Sciences, Nanjing, 210014 China; 5 Faculty of Life Science, University of Manchester, Michael Smith Building, Oxford Road, Manchester, M13 9PT UK; 6 Research Institute of tropical forestry, Chinese Academy of Forestry, Guangzhou, 510520 China; 7 Boyce Thompson Institute for Plant Research, Ithaca, 14853 USA

**Keywords:** Eucalyptus, TPX2 family proteins, Phylogenetic analysis, Expression profiling, Subcellular localization, Organ twisting

## Abstract

**Background:**

The Xklp2 (TPX2) proteins belong to the microtubule-associated (MAP) family of proteins. All members of the family contain the conserved TPX2 motif, which can interact with microtubules, regulate microtubule dynamics or assist with different microtubule functions, for example, maintenance of cell morphology or regulation of cell growth and development. However, the role of members of the TPX family have not been studied in the model tree species *Eucalyptus* to date. Here, we report the identification of the members of the TPX2 family in *Eucalyptus grandis* (Eg) and analyse the expression patterns and functions of these genes.

**Results:**

In present study, a comprehensive analysis of the plant TPX2 family proteins was performed. Phylogenetic analyses indicated that the genes can be classified into 6 distinct subfamilies. A genome-wide survey identified 12 members of the *TPX2* family in the sequenced genome of *Eucalyptus grandis*. The basic genetic properties of the TPX2 family in Eucalyptus were analysed. Our results suggest that the TPX2 family proteins within different sub-groups are relatively conserved but there are important differences between groups. Quantitative real-time PCR (qRT-PCR) was performed to confirm the expression levels of the genes in different tissues. The results showed that in the whole plant, the levels of *EgWDL5* transcript are the highest, followed by those of *EgWDL4*. Compared with other tissues, the level of the *EgMAP20* transcript is the highest in the root. Over-expression of *EgMAP20* in Arabidopsis resulted in organ twisting. The cotyledon petioles showed left-handed twisting while the hypocotyl epidermal cells produced right-handed helical twisting. Finally, EgMAP20, EgWDL3 and EgWDL3L were all able to decorate microtubules.

**Conclusions:**

Plant TPX2 family proteins were systematically analysed using bioinformatics methods. There are 12 TPX2 family proteins in *Eucalyptus*. We have performed an initial characterization of the functions of several members of the *TPX2* family. We found that the gene products are localized to the microtubule cytoskeleton. Our results lay the foundation for future efforts to reveal the biological significance of TPX2 family proteins in *Eucalyptus*.

**Electronic supplementary material:**

The online version of this article (doi:10.1186/s12864-016-3303-0) contains supplementary material, which is available to authorized users.

## Background

Microtubules (MTs) are hollow tubular structures formed by non-covalent bonds between 13 tubulin proto-fibrils. These 22–25 nm diameter proto-fibrils are composed of α-tubulin and β-tubulin heterodimers concatenated end-to-end. γ-tubulin, which is not a structural component of microtubules, guides the tubulin dimer during MT assembly. Some MT structures are relatively stable due to the role of MT binding proteins and enzyme modifications. In many physiological activities, MTs regulate their own dynamic changes through microtubule-associated proteins (MAPs). MAPs are also involved in other MT functions, such as maintenance of cell morphology and structure, participation in cytoplasmic streaming, membrane transport, determining the position of the organelles, regulation of signal transduction, controlling cell polarity growth, cell wall construction and so on [1–4].

MAPs are a class of proteins that specifically bind to the MT cytoskeleton and modulate their dynamic assembly processes and structures, thereby affecting their function. Compared to the MAPs in animal systems, research on plant MAPs started later. It was not until the 1980’s that first plant MAPs were discovered and their cellular functions were analysed. The MAPs contain at least one outwardly prominent structural domain which stretches out to MTs and interacts with other cell components (e.g. MT bundle, intermediate fibre and plasma membrane). In recent years, a number of new functional studies on plant MAPs have been reported, such as MAP65, MAP70 and MAP18, MAP20 etc. [5–12]. These results showed that the plant MAPs can regulate plant MT dynamics and organization, as well as the connection between MTs and other cellular structures, thereby playing a role in plant cell morphology and differentiation, plant growth and development, adaptation to diverse physiological processes [5, 13–18]. All these important biological roles mean that MAPs and how they regulate the MT activity is a focus of considerable current research.

TPX2 family proteins have a highly conserved TPX2 domain (Pfam: PF06886) which was first found in a kinesin like protein, Targeting Protein for Xklp2 (TPX2) from *Xenopus laevis*. The primary structure of TPX2 is conserved among vertebrates and higher plants. Recently, studies have reported that several plant TPX2 family proteins, such as TPX2, WAVE DAMPENED 2 (WVD2), WAVE DAMPENED 2 LIKE (WDL) 1, 2 and 3 and MAP20, take part in plant development [12, 19–23]. In plants, TPX2 has two Aurora binding domains, two nuclear localization signal (NLS) motifs, a nuclear export signal (NES) motif, a TPX2-importin domain (Pfam: PF12214), a TPX2 domain (pfam: PF06886), a coiled-coil domain and two MT binding regions [22, 24]. The TPX2 N-terminal Aurora binding domains allow TPX2 to bind Aurora kinases and regulates spindle formation, while the C terminus binds microtubules and affects cell division [24]. TPX2 is a new spindle component protein in vertebrates. It distributes to the core of cells in the S and G2 phase of the cell cycle and plays an important role in the process of mitotic spindle formation. During interphase, TPX2 is localized to the nucleus, and in the G0/M phase, the nuclear envelope breakdown leads to the release of TPX2 and the start of spindle assembly during prophase. During mitosis, TPX2 is localized to the spindle microtubules, and in late anaphase it is completely degraded [21]. In the Ran GTPase complex, TPX2 is released from the importin complex, interacts with Aurora kinases and combines with microtubules, and controls the cell division [19, 25].

AtWVD2 is a conserved, highly hydrophilic protein [26] which can interact with microtubules to promote MT bundling. AtWVD2 plays an important role in polar cell elongation. The roots and etiolated hypocotyls of plants overexpressing WVD2 displayed twisting in a right handed helical manner, but rosette leaf petioles twisted in opposite direction [26]. These helical twisted growth phenotypes may result from an interplay between cortical microtubules and cellulose microfibrils. Moreover, the loss of anisotropic cell elongation results in the generation of short and robust organs. In addition, the arrangement of cortical microtubules in the root epidermal cells of these plants is also changed [26]. Arabidopsis WDL3, another TPX2 family member, functions in hypocotyl cell elongation in response to light via an ubiquitin-26S proteasome–dependent pathway. AtWDL3 overexpression and an RNAi downregulated line showed shorter and longer hypocotyl cells than wild type, respectively [23]. Arabidopsis WDL5 as a microtubule-stabilizing protein is involved in ethylene mediated etiolated hypocotyl cell elongation by altering the organization and stability of cortical microtubules [26].

Apart from a role in root development, TPX2 protein family members also function in cell wall biosynthesis. Hybrid aspen (*Populus tremula × tremuloides*) MAP20 (PttMAP20) was identified as being abundant in secondary cell wall forming woody tissues. PttMAP20 was shown to bind the cellulose inhibiting drug 2,6-dichlorobenzonitrile (DCB), implying its importance for cellulose biosynthesis in the secondary cell walls. Overexpression of MAP20 resulted in right handed helical twisting of epidermal cells and cotyledon petioles [12].

Given the important roles of TPX2 family proteins in plant development, a phylogenetic analysis of TPX2 family proteins from a large number of plant species is valuable. In this study, we report global phylogenetic analysis of plant TPX2 proteins and their classification into 6 groups. We also discuss 12 members of the TPX2 family in *Eucalyptus grandis*, as well as their expression profiling in six different tissues. The Eucalyptus *TPX2* genes exhibited diverse expression patterns, suggesting their functional divergence. We also carried out preliminary functional analysis of selected Eucalyptus *TPX2* genes. Our results provide the basis for further investigation into the roles of these candidate genes in cell division, growth and development of *Eucalyptus*.

## Results and discussion

### Bioinformatic analysis of plant TPX2 family proteins

**Fig. 1:**
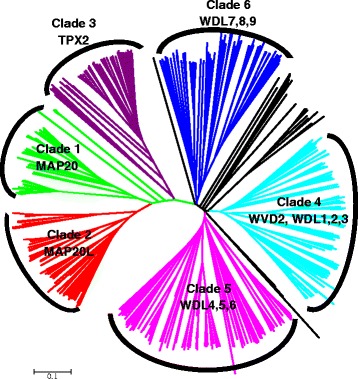
Phylogenetic relationship among Plant TPX2 proteins. A total of 763 protein sequences were identified by PFAM containing the TPX2 domain (PF06886). Of these, 574 sequences were plant (Viridiplantae) sequences. The plant sequences were aligned using ClustalX2.1 and a bootstrapped neighbour joining tree was constructed. Six main clades were identified (colored nodes) and named after the Arabidopsis members in each clade. Some sequences which fall between clades (black nodes) were not used for the domain analysis in Fig. 2. These sequences still contain the TPX2 domain

To identify putative plant TPX2 family proteins, the conserved TPX2 (PF06886) domains were searched from the PFAM database. The database currently holds a total of 763 sequences from 188 species. Based on the presence of other domains, these sequences can be classified into 17 different domain architectures (Additional file 1: Figure S1). We extracted all 763 sequences from PFAM database and identified that 574 of these sequences were from 45 different species of plants (Viridiplantae) (Additional file 2: Table S1). These sequences include multiple splice variants for different genes. The plant TPX2 sequences were aligned using ClustalX2.1 and a phylogenetic tree was produced (Fig. 1).

**Fig. 2:**
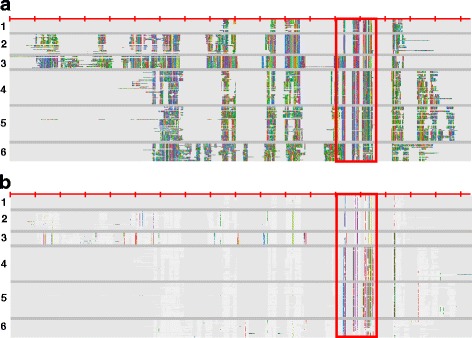
Overall structure of plant TPX2 proteins. An alignment of TPX2 proteins from all 6 clades (clade number on the left of the figure) described in Fig. 1 was used to show the overall size and structure of the proteins. The alignment was coloured in AliView to show physiochemical properties of the amino acids. **a** and **b** both show the same alignment coloured to highlight either all amino acids (**a**) or majority rule consensus residues (**b**)

The phylogenetic tree reveals 6 main clades. Clade 1 contains MAP20, the protein first reported from wood forming tissues of *Populus* [12] and its homologues from different plant species [27]. Clade 2 does not contain any protein that has been previously described. Since these are closest to MAP20 clade, we name this clade as MAP20L. However, it should be considered that these proteins are much larger than MAP20 (Fig. 2). Clade 3 contains the best studied plant TPX2 protein, the AtTPX2 [22]. Clades 4–6 contain WVD2 and WDL proteins [23, 26, 28, 29]. Some of the sequences fell in between the clades. While these sequences contain the TPX2 domain and are mostly from lower plants, these are sufficiently different to produce large insertions in the global alignment of plant TPX2 proteins. Hence, for some of the analyses presented here, these sequences were not used. A full list of number of sequences in each clade is presented on Additional file 3: Table S2.

A global alignment of plant TPX2 proteins from 6 main clades (Fig. 2) reveals that the clade 1 (MAP20) proteins are the smallest of the TPX2 proteins while the clade 2 (MAP20L) and clade 3 (TPX2) are the largest. Proteins in clades 4–6 (WDLs) are of intermediate size. An analysis of sequence conservation across various clades (Fig. 2b) reveals that there is little or no sequence conservation among the proteins outside of the TPX2 domain. It is likely that members of different clades would serve different functions. They all contain the TPX2 domain which has been shown to bind the microtubules [20, 22]. It is likely these proteins will bind to microtubules and at the same time interact with other proteins to play an important role in a variety of plant developmental processes involving in a role for microtubules.

**Fig. 3:**
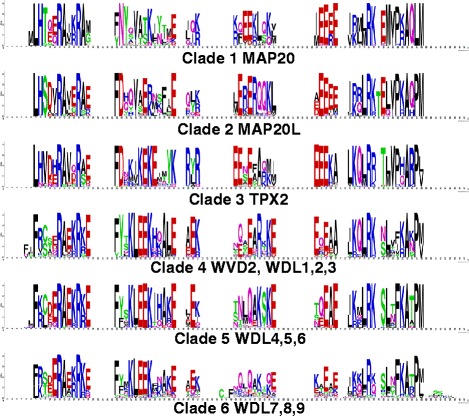
Analysis Plant TPX2 domains. The plant (Viridiplantae) TPX2 proteins (574 sequences) were categorised into 6 main groups. An alignment of the TPX2 domain (PF06886) sequences was then obtained from PFAM database. TPX2 domain sequences for each individual clade were fed into the online server WebLogo to create the sequence logos to highlight the conserved residues for each group

An analysis of extracted TPX2 domains from all plant TPX2 proteins reveals that there are some differences in the sequences between the different clades. The most apparent of these differences is the presence of a KLEEK motif in the clades 4–6. This motif is absent in proteins from clades 1–3 (Fig. 3).

In the PFAM database, there were 31 sequences from Arabidopsis (At) and 19 sequences from Eucalyptus (Eg). Since these sequence sets include the splice variants, the fully sequenced genomes available at Phytozome were scanned to identify 15 TPX2 loci in Arabidopsis and 12 loci in Eucalyptus (Additional file 4: Table S3). The Eucalyptus proteins were named based on known Arabidopsis homologues. A similar nomenclature approach has been used previously for CESA proteins [30]. The final list of Eucalyptus loci included EgMAP20 (Clade 1), EgMAP20L (Clade 2); EgTPX2 (Clade 3); EgWDL1, EgWDL3 and EgWDL3L (Clade 4); EgWDL4, EgWDL5 and EgWDL6 (Clade5) and EgWDL7, EgWDL8 and EgWDL8L (Clade 6).

### Genetic properties of Eucalyptus *TPX2* family genes

**Fig. 4:**
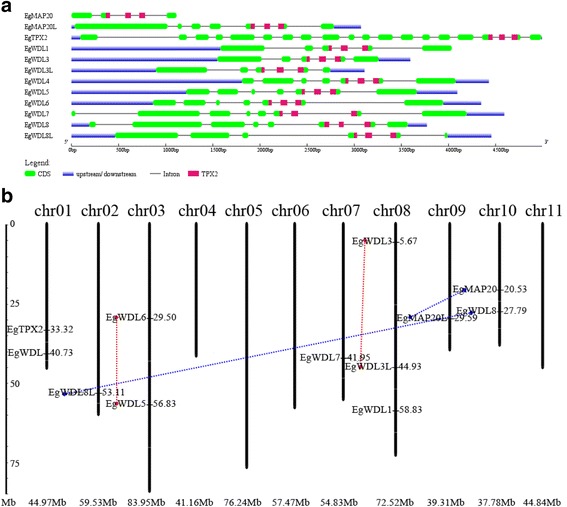
Genetic properties of Eucalyptus TPX2 proteins. **a** Exon/intron structures of Eucalyptus TPX2 family genes. Green boxes represent the CDS regions while the blue boxes represent the untranslated regions. Introns are indicated by black lines. Location of the TPX2 domain is indicated in pink. The sizes of exons and introns can be estimated using the scale at bottom. **b** Chromosomal locations of Eucalyptus TPX2 family genes. Tandem duplications were connected by dashed red dotted lines. Non-tandem duplications were connected by blue dotted lines

The Eucalyptus *TPX2* family gene structures were compared using online software GSDS 2.0 [31]. The core TPX2 domain is usually spread across three exons in all Eucalyptus TPX2 genes, which are followed by another exon downstream (Fig. 4a). EgMAP20 is the smallest of the 12 Eucalyptus TPX2 genes and has only one more exon on the 5′ side of the TPX2 domain. EgMAP20L on the other hand has four exons upstream of TPX2 domain, making a total of 8 exons for this gene. EgTPX2 is the largest of the 12 genes and comprises of a total of 20 exons and 19 introns. The remaining genes, 9 WDL genes, have between 2 and 5 exons upstream of the TPX domain making a total of 6–9 exons in these genes (Fig. 4a).

Modern plant genome diversity has evolved via gene deletions, small-scale duplications, partial rearrangements and large chromosomal fragment duplications, which significantly impacted the expansion in gene family members [32]. The 12 Eucalyptus *TPX2* family genes showed random distribution along *Eucalyptus* chromosomes on the basis of the chromosome information provided by Phytozome (http://www.phytozome.net/). The 12 *TPX2* family genes are distributed on 7 of the 11 Eucalyptus chromosomes (Fig. 4b). Based on the phylogenetic tree, we linked 4 pairs of the paralogous *TPX2* family genes (red and blue dotted line pairs in Fig. 4b). *EgWDL5/EgWDL6* and *EgWDL3/EgWDL3L* are the tandem duplication pairs on chromosome 3 and 8 respectively. However the paralogous duplication pairs, *EgMAP20/EgMAP20L* and *EgWDL8/EgWDL8L* are located on chromosomes 10/9 and 10/2 respectively (Additional file 5: Table S4). The conservation and micro-colinearity of Eg*TPX2* family genes show symbiotic evolution and suggest a common origin of these genes. Together, the diverse duplication events contributed to the complexity of *TPX2* gene family in the *Eucalyptus* genome.

The synonymous (Ks) and non-synonymous (Ka) substitution rates ratios (Ka/Ks ratio) were used to analyse the TPX2 gene pairs. The synonymous substitutions do not change the amino acid sequence and are subjected to a lower selection pressure. On the other hand, non-synonymous substitutions change the amino acid sequence which might lead to harmful mutations and hence are under a higher selection pressure. When Ka/Ks is close to 1, it indicates evolution under neutral selection. A Ka/Ks ratio of < 1 indicates that those genes undergo a purifying (stabilizing) selection while Ka/Ks > 1 at specific sites indicates genes that are under positive selection. In most cases, the Ka/Ks ratio is less than 1 due to the purifying selection. However, when the diversifying selection exists, the Ka/Ks of the allele will increase, even significantly higher than 1 [33]. Additional file 5: Table S4 shows that the Ka/Ks ratios of all 4 duplicated pairs were all less than 0.9, indicating purifying selection.

### Functional analysis of Eucalyptus *TPX2* family genes

#### Expression analysis of *TPX2* family genes in *Eucalyptus*

**Fig. 5:**
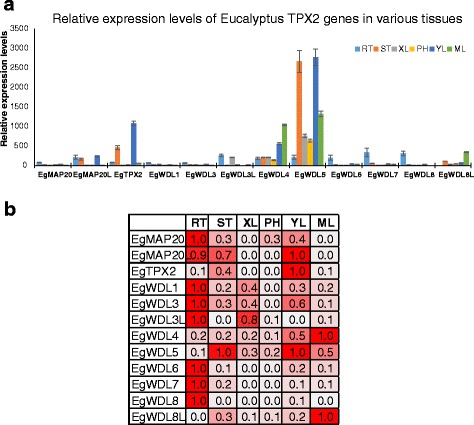
Expression analysis of Eucalyptus TPX2 family genes in different tissues. **a** Expression of all 12 Eucalyptus TPX2 family genes was quantified by quantitative reverse-transcription polymerase chain reaction (qRT-PCR) in vegetative tissues (ST, shoot tips; YL, young leaves; ML, mature leaves; PH, phloem; XL, xylem; RT, roots). The average expression of each gene was calculated relatively to the first biological replicate of roots ± standard error (SE) (*n* ≥ 3). **b** Relative expression of each Eucalyptus TPX2 genes was normalised to its highest expression across various tissues. This will make the highest level of each gene as 1

The expression patterns of genes can provide useful clues to their function. To identify the expression patterns of *TPX2* family genes in plants, 6 different tissues of *Eucalyptus grandis* - shoot tips (ST), young leaves (YL), mature leaves (ML), phloem (PH), xylem (XL), roots (RT)) were analysed by quantitative RT-PCR (Fig. 5a). In general, expression of *EgWDL5* was the highest among the Eucalyptus TPX2 genes followed by *EgWDL4* and *EgTPX2*. All other genes were expressed at comparatively lower levels.

To compare relative expression levels of each gene across multiple tissues, we calculated the normalised expression values for each gene (Fig. 5b). Seven out of 12 Eucalyptus genes (*EgMAP20, EgWDL1, EgWDL3, EgWDL3L, EgWDL6, EgWDL7* and *EgWDL8*) had highest expression in roots. *EgMAP20L* and *EgTPX2* had highest levels in young leaves while *EgWDL4* and *EgWDL8L* had highest levels in mature leaves. While the expression of *EgWDL5* was the highest in the shoot tips and young leaves (Fig. 5b). The observation that most of TPX2 genes had their highest levels in root indicated that they may be functionally important in root development.

#### Subcellular localization of Eucalyptus TPX2 proteins

**Fig. 6:**
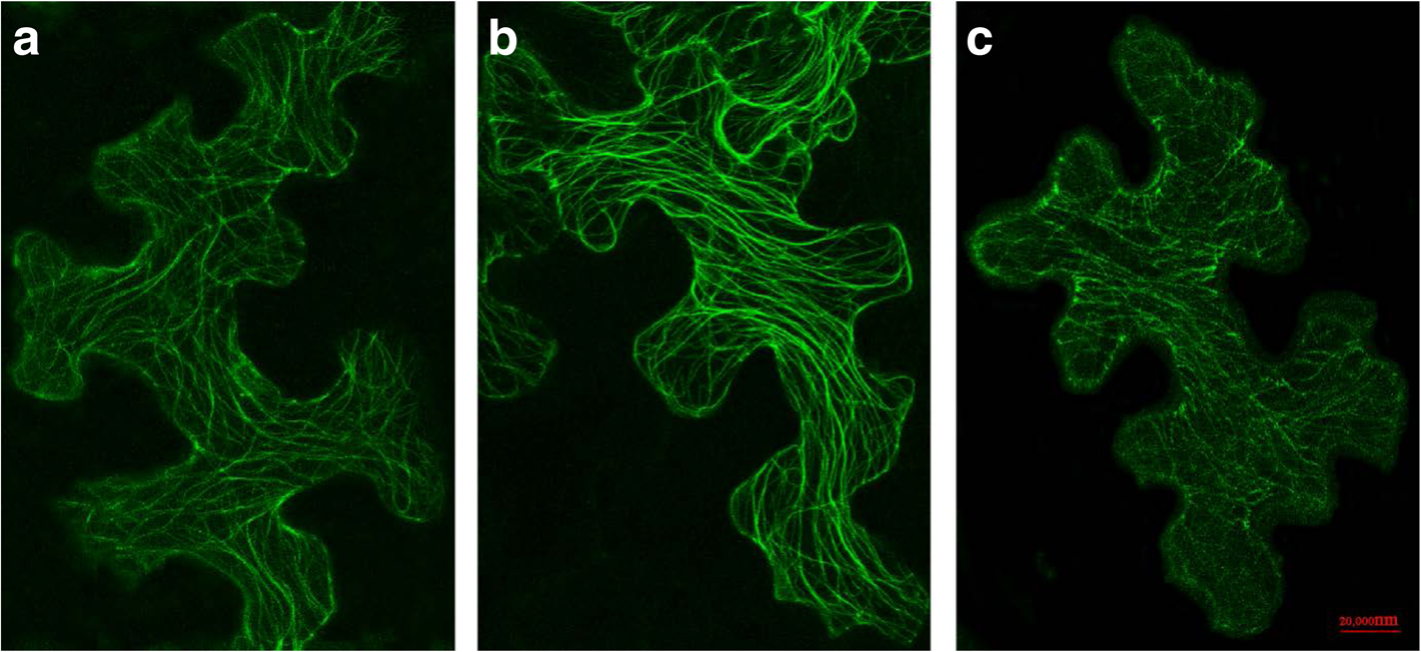
Subcellular localizations of three Eucalyptus TPX2 family proteins. Confocal images of tobacco epidermal leaf cells expressing EgMAP20-YFP (**a**), EgWDL3-YFP (**b**) and EgWDL3L-YFP (**c**). Scale bar = 20 μM

Despite their variable domain structures and expression patterns, one common feature of TPX2 family proteins is that they are all likely to be MAPs and have MT binding activity. To investigate their MT binding ability in vivo, we examined the subcellular localization of a selection of Eucalyptus TPX2 proteins. Coding sequences (CDS) of EgMAP20, EgWDL3 and EgWDL3L were fused to C-terminal Yellow Florescence Protein (YFP) tags (EgMAP20-YFP, EgWDL3-YFP and EgWDL3L-YFP) and the constructs were transiently expressed in tobacco leaf epidermal cells. Confocal microscopy observations of the EgMAP20-YFP, EgWDL3-YFP and EgWDL3L-YFP florescence signals form net-like structures throughout the cell (Fig. 6) indicating that these proteins are all distributed along the MT cytoskeleton. Previously, poplar PttMAP20-YFP has been shown to distribute along microtubules [12]. In addition, PttMAP20 was strongly up-regulated during secondary cell wall synthesis in hybrid aspen and tightly co-regulated with CESA genes [12]. WVD2, WDL1 and WDL3 have also been localized to cortical microtubules [23, 28]. TPX2 has been shown to localize to the cortical microtubules in interphase, and it may decorate other MT arrays during other stages of plant cell division (preprophase band, spindle, phragmoplast) [22]. TPX2 family proteins contain a conserved MT binding domain, the TPX2 domain, which plays an important role in the organization of the MT arrays, cell growth and the regulation of cell division [34]. Taken together, previous studies and our own observations suggest that EgMAP20, EgWDL3 and EgWDL3L are MT binding proteins like their homologues from Populus and Arabidopsis.

#### Phenotypic observation of transgenic plants

##### EgMAP20 overexpression in Arabidopsis leads to organ twisting

**Fig. 7:**
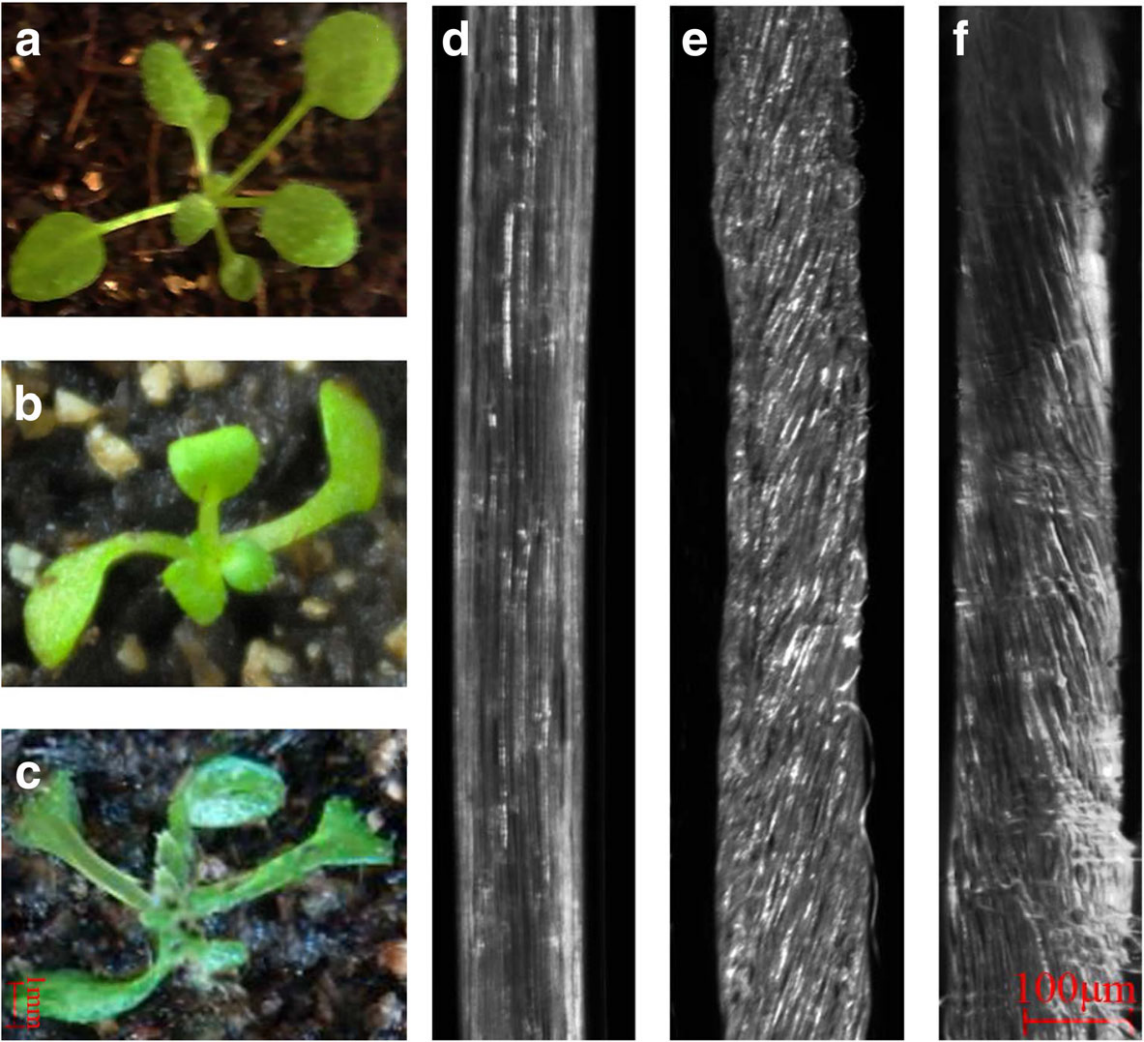
Phenotypic analysis of transgenic lines of Arabidopsis overexpressing EgMAP20. **a-c**, 12-day-old wild-type (**a**) and transgenic OE-EgMAP20 (**b** and **c**) Arabidopsis seedlings are shown. The latter showing right-handed (**b**) or left-handed (**c**) twisting of cotyledon petioles. **d-f**, Etiolated hypocotyls from 3-day-old wild-type (**d**) and two independent overexpressing EgMAP20 lines (**e** and **f**). Helical right-handed (**e**) or left-handed (**f**) twisting of epidermal cells was observed in the two transgenic lines

To understand the effect of the TPX2 family proteins on plant growth and development, p35S:*EgMAP20* overexpressing transgenic Arabidopsis plants were analysed. The cotyledons of 12-day-old seedlings overexpressing *EgMAP20* developed left/right handed twisting of epidermal cells while etiolated hypocotyls of 3-days-old seedlings showed right/left-handed twist (Fig. 7a-f).

Similar twisting phenotypes with overexpression of TPX2 proteins from other species have been reported previously. Overexpressing *PttMAP20* in Arabidopsis caused cotyledon petioles with left-handed helical twisting and hypocotyl epidermal cells with a right-handed helical twist [12]. Overexpression of MT bundling proteins, WVD2 and WDL1 in Arabidopsis led to a right hand deviation of roots and left hand skewing of cotyledon petioles [26] but left-handed twisting of the rosette leaves. During organ twisting, the anisotropy of cell elongation is lost, leading to generation of short and robust cells. In addition, cortical MT arrays of root epidermal cells are also changed [28]. Overexpression of *TPX2* caused the random organization of cortical microtubules and root right handed shift [25]. However, overexpression of the MT depolymerising protein MAP18 produced a left handed twist of hypocotyl epidermal cells [11].

The growth of plant cells involves an increase in cell volume, which can be achieved by cell expansion or elongation. Both of these two processes occur in a specific area of the cell surface causing the changes in cell morphology. Three kinds of MT arrays participating in cell morphogenesis exist in plants, including cortical MTs, preprophase bands and phragmoplast MTs. The cortical MTs during interphase are involved in controlling the arrangement of cellulose microfibrils in the cell wall and thereby determining the direction of cell elongation [35]. It is plausible that MAP20 plays a role in cell elongation leading to directional skewing. To understand why cotyledon and hypocotyl cells of *EgMAP20* overexpression plants are sometimes left/right spiral, further experiments are needed.

##### EgWDL3L overexpression in Arabidopsis affects growth

**Fig. 8:**
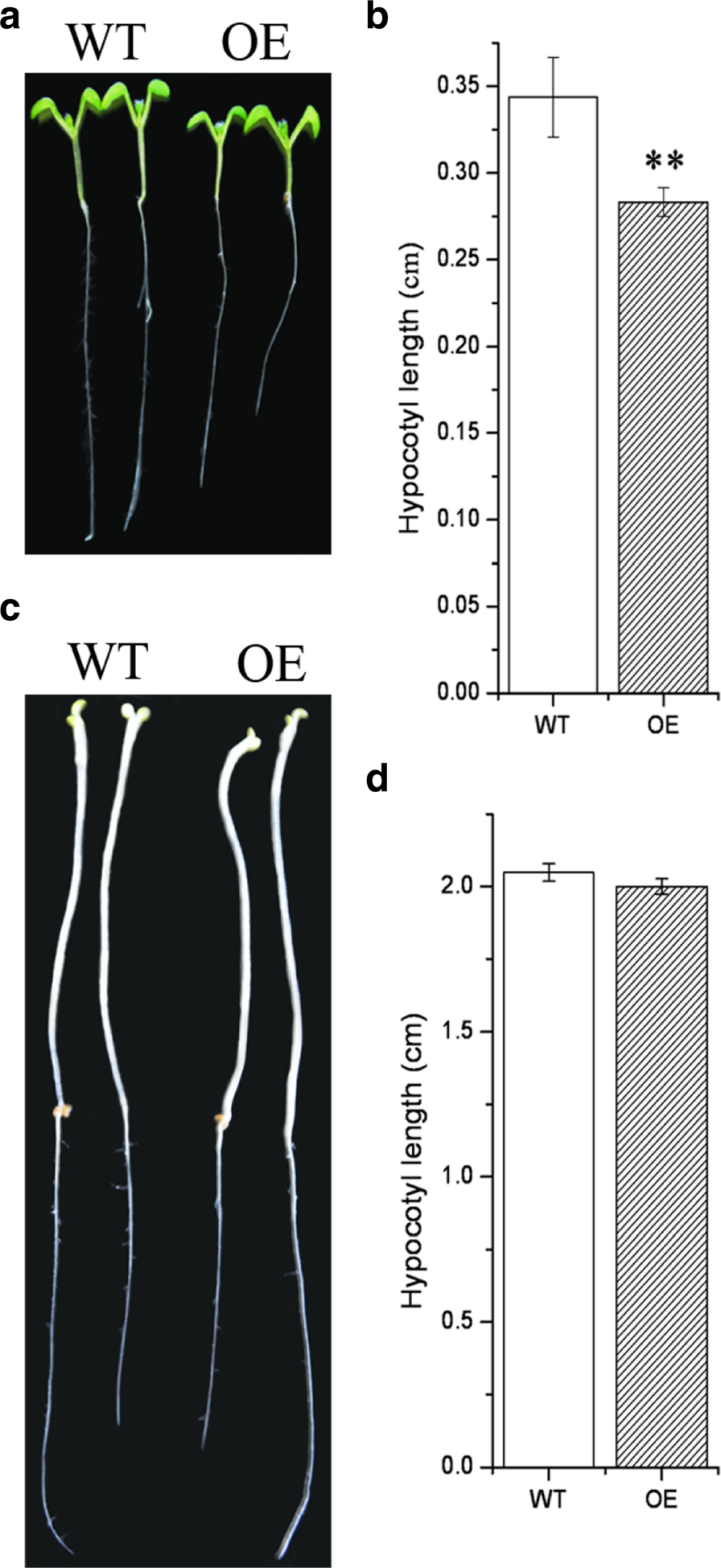
Effect of EgWDL3L on the hypocotyl cell elongation. **a** and **b** the hypocotyl length of the seedlings grown under light. **P < 0.01 with *t*-test, *n* >30, error bars represent the mean ± SD. **c** and **d** Etiolated hypocotyl length. *n* >30, error bars represent the mean ± SD

The over-expression (OE) of EgWDL3L in Arabidopsis on the other hand did not lead to any helical twisting. There was no difference between the wild type and EgWDL3L OE lines grown in dark. However, the hypocotyls were shorter for the light grown seedlings (Fig. 8). Previous studies have shown that AtWDL3 is a negative regulator of the hypocotyl cell elongation and acts in a light dependent manner. In light, AtWDL3 is relatively stable and promotes MTs to form a longitudinal arrangement, thereby inhibiting hypocotyl cell elongation. However, in the dark, AtWDL3 is degraded via the 26S proteasome pathway, thereby removing inhibition of hypocotyl cell elongation [23]. Since EgWDL3L and AtWDL3 are closely related and are both part of Clade 4, EgWDL3L like AtWDL3 may also be a negative regulator of cell elongation.

## Conclusion

We performed a comprehensive analysis of phylogeny of plant TPX2 proteins from the PFAM database. Expression analysis of 12 Eucalyptus *TPX2* genes suggested that most of *TPX2* genes might mainly be functionally important during root development. Furthermore, overexpression of EgMAP20 and EgWDL3L leads to a range of twisting phenotypes that are indicative of modulation of MT arrays and hence suggests that these proteins are indeed MAPs. MAPs modulate a variety of developmental activity in plants and previous studies have shown that members of TPX2 family are involved in multiple processes in plant development, including root development, embryogenesis and formation of secondary cell walls. This research forms an important foundation for further investigations into the role of TPX2 proteins in Eucalyptus growth and development.

## Methods

### Sequence retrieval and phylogenetic analysis

To identify plant TPX2 family, all TPX2 domain (PF06886) containing proteins were extracted from the PFAM database (http://pfam.xfam.org/family/PF06886#tabview=tab1). This page listed 763 sequences from 188 different species. After obtaining uniprot IDs for 763 sequences from PFAM, full length protein sequences were retrieved from uniprot database (http://www.uniprot.org/). By using the associated taxonomic information, 574 plant sequences were identified. All plant sequences were aligned with ClustalX2.1 [36] and a bootstrapped neighbour joining tree was produced. Further rendering of the tree and subtree-colouring was performed in MEGA6 suite [37] to produce Fig. 1. Our data for the phylogenetic tree was deposited into TreeBase at the link https://treebase.org/treebase-web/search/study/summary.html?id=19741. Aligned full length sequences were coloured in AliView [38] and alignment images were exported to produce Fig. 2.

For analysing the domain motifs, pre-aligned TPX2 domains were downloaded from PFAM website and sequences were classified into 6 groups based on Fig. 1. Sequence conservation logos shown in Fig. 3 were drawn with WebLogo [39].

To extract all TPX2 proteins from Arabidopsis and Eucalyptus, BLAST searches against *Eucalyptus grandis* (v1.1) [40], *Arabidopsis thaliana* (TAIR 10), genomes at Phytozome database (http://www.phytozome.net/) were performed. Initially 21 sequences for Eucalyptus and 25 sequences for Arabidopsis were obtained. After removing the splice variants and keeping one representative transcript, 12 Eucalyptus and 15 Arabidopsis sequences were retained.

### Gene structure analysis

The exon and intron structures of Eucalyptus *TPX2* family genes were analysed using Gene Structure Display Server (GSDS 2.0,http://gsds.cbi.pku.edu.cn/) [31].

### Chromosomal location

Chromosome location images were generated by using the MapInspect software to localize Eucalyptus *TPX2* family genes. Tandem duplications were connected by dashed red dotted lines. Non-tandem duplications were connected by blue dotted lines. The ratio between non-synonymous and synonymous nucleotide substitutions (Ka/Ks) was calculated using DNAsp5 software (http://www.ub.edu/dnasp/) [41] for selected pairs of homologous genes.

### Plant material cultivation

*Eucalyptus grandis* plants were grown on local soil for 10 months under outdoor conditions. Arabidopsis and *Nicotiana benthamiana* plants were grown in an incubator with following conditions: 22 °C, 16 h light/8 h dark, 60% relative humidity, light intensity of 100 ~ 120 μmol · m^−2^ · S^−1^. The soil mixture contained 3 parts peat soil (Holland) and 1 part vermiculite.

### Quantitative real-time PCR

The EASY spin plant RNA Kit (Aidlab biotech, Beijing, China) was used to extract total RNA from six different tissues of Eucalyptus (ST, shoot tips; YL, young leaves; ML, mature leaves; PH, phloem; XL, xylem; RT, roots). Specific RT-PCR primers for 12 Eucalyptus *TPX2* family genes were designed with premier primer 5 (Additional file 6: Table S5). A total of 1 μg RNA per sample was reverse transcribed into cDNA with the PrimeScript™ II reverse transcription kit (TaKaRa Biotech. Co. Ltd., Dalian, China). The cDNA samples were diluted 1:10 with nuclease-free water prior to the qRT-PCR analyses. qRT-PCR was performed in quadruplicates using the SYBR Premix Ex Taq™ II Kit (TaKaRa, Dalian, China) on a Roche Light Cycler® 480 (Roche Ltd. Mannheim, Germany) according to the manufacturer’s instructions. The PCR reaction was performed in a total volume of 20 μl, containing 10 μl of 2 × SYBR Premix, 2 μl of cDNA template, and 0.4 μl of each specific primer to a final concentration of 100nM. The reactions were performed using the following conditions: initial denaturation 95 °C for 30 s and 40 cycles of amplification - 95 °C for 5 s, 58 °C for 30 s and 72 °C for 18 s and final extension 72 °C for 2 min. Melting curve analysis was performed at 95 °C for 5 s, 58 °C for 30 s, 95 °C continuous; cooling 40 °C for 30 s. *EgCDK8* was used as the reference gene. The qRT-PCR data were analysed by Livak 2^-ΔΔCT^.

### Transient expression and imaging

Plant DNA Kit HP (OMEGA) was used to extract the DNA of Eucalyptus which was used as template to amplify *EgMAP20*, *EgWDL3* and *EgWDL3L*. Recombinant plasmids pEarleygate101-*EgWDL3*-YFP and pEarleygate101-*EgWDL3L*-YFP were constructed using the Gateway recombination reactions. We used the ClonExpress™II One Step Cloning Kit (Vazyme Biotech Co., Ltd, China) to construct binary expression vector pGWB2-*EgMAP20*-YFP. Laser scanning confocal microscope was used to observe the fluorescence in the tobacco leaf epidermal cells 48 to 72 h after infiltration [42]. Images were collected by 780/7 live Zeiss laser scanning confocal microscope (Carl Zeiss AG, Germany) with excitation at 514 nm, scanning at 520–555 nm. A 63× objective lens was used. The images were collected at 1024 × 1024 pixel resolution.

### Transgenic phenotype observation

The binary expression vectors pGWB2-*EgMAP20* (*p35S:EgMAP20*) and pEarleygate100-*EgWDL3L* (*p35S:EgWDL3L*) were constructed using the Gateway system, transformed into Agrobacterium C58 and used to transform wild-type Arabidopsis using the floral dip method [43]. T1 seed was harvested and EgMAP20 transgenic lines were selected on MS solid medium with kanamycin 50 μg/ml while EgWDL3L lines were screened using the herbicide BASTA. Positive seedlings were transplanted on soil. T2 generation seed of EgMAP20 and EgWDL3L and wild type seeds were plated on MS agar plates. After dark culture for 3 days, we observed hypocotyl cells of EgMAP20 seedlings under a stereomicroscope (Leica). After 12 days, EgMAP20 cotyledons were observed. Light and dark culture after 7 days, we observed EgWDL3L seedling hypocotyls, took pictures with a Nikon D300s camera and measured lengths using Image J.

## Additional files

Additional file 1: Figure S1. Domain architectures of PFAM TPX2 proteins. All TPX2 domain containing proteins in PFAM can be classified into 17 different domain architectures. This figure was collected from PFAM website as it is. All proteins have the PF06886 (green blob). (PPTX 873 kb)
Additional file 2: Table S1. TPX2 proteins in plants (Viridiplantae). 574 of these Plant TPX2 proteins were from 45 different species of plants (Viridiplantae) and were used for producing the phylogenetic tree shown in Fig. 1. These sequences were classified into 6 groups (clade 1–6). (XLSX 325 kb)
Additional file 3: Table S2. Distribution of PFAM plant TPX2 proteins in various clades. A complete list of number of sequences in each clade (clade 1–6) is presented. (XLSX 17 kb)
Additional file 4: Table S3. Genome-wide analysis of Arabidopsis and Eucalyptus TPX2 genes. Gene name, locus ID and the clade number in the phylogenetic tree (Fig. 1) are shown. (XLSX 10 kb)
Additional file 5: Table S4. The Ka/Ks ratios and Evolutionary selection of paralogous TPX2 family proteins. Ks: number of synonymous substitutions per synonymous site; Ka: number of nonsynonymous substitutions per nonsynonymous site. When Ka/Ks = 1, neutral evolution; Ka/Ks < 1, purifying selection; Ka/Ks > 1, diversifying selection. Genes in duplicated pairs are in tandem duplication (Tandem), in homeologous chromosomes (Homeologous) or were simply paralogous. (XLSX 11 kb)
Additional file 6: Table S5. Primer sequences for 13 genes for RT-qPCR analysis. EgCDK8 was used as a reference gene while others are Eucalyptus TPX2 family genes. (XLSX 10 kb)

## Abbreviations

CDS: Coding Sequence
DCB: 2,6-dichlorobenzonitrile
Eg: *Eucalyptus grandis*
GSDS: Gene Structure Display Server
Ka: Nonsynonymous mutation rate
Ks: Synonymous mutation rate
MAP: Microtubule-associated protein
ML: Mature leaves
MT: Microtubule
NES: Nuclear export signal
NLS: Nuclear localization signal
OE: Over-expression
PCR: Polymerase chain reaction
PH: Phloem
qRT-PCR: Quantitative real-time RT-PCR
RT: Roots
ST: Shoot tips
TPX2: Targeting protein for Xklp2
WDL: Wave Dampened 2 LIKE
WVD2: Wave Dampened 2
XL: Xylem
YFP: Yellow Florescence Protein
YL: Young leaves
