# Supplementary figures and images for: Genome-wide analysis of the TPX2 family proteins in Eucalyptus grandis
Source: BMC Genomics. 2016 Nov 24;17:967. doi: 10.1186/s12864-016-3303-0 (PMC5122032; doi:10.1186/s12864-016-3303-0)

## Slide 1
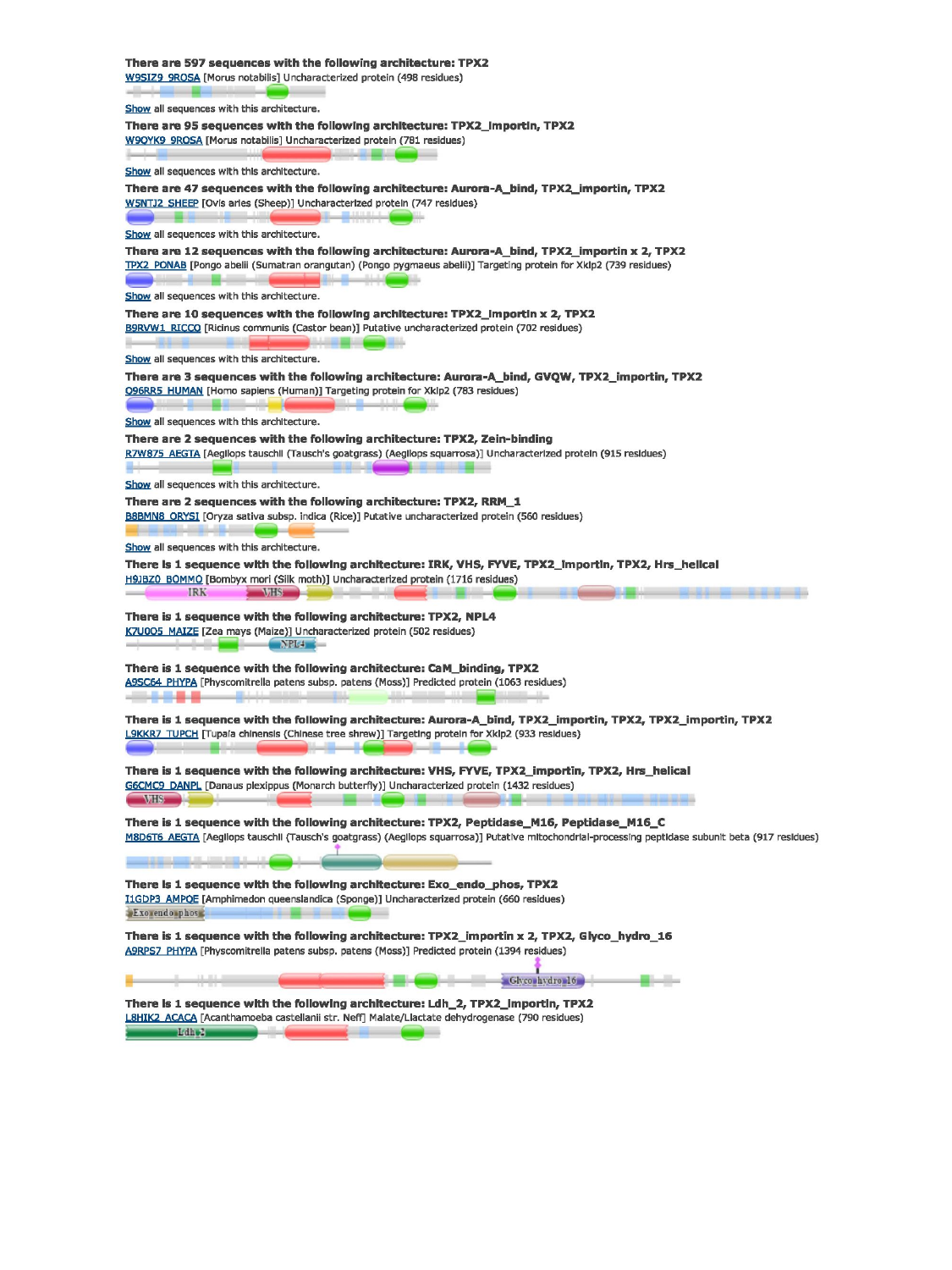

Supplement: Additional file 1: Figure S1. — Domain architectures of PFAM TPX2 proteins. All TPX2 domain containing proteins in PFAM can be classified into 17 different domain architectures. This figure was collected from PFAM website as it is. All proteins have the PF06886 (green blob). (PPTX 873 kb) [file 12864_2016_3303_MOESM1_ESM.pptx]
